# Ki67 as a prognostic factor of craniopharyngioma’s recurrence in paediatric population

**DOI:** 10.1007/s00381-020-04519-4

**Published:** 2020-02-07

**Authors:** Elżbieta Moszczyńska, Monika Prokop-Piotrkowska, Agnieszka Bogusz-Wójcik, Wiesława Grajkowska, Sylwia Szymańska, Mieczysław Szalecki

**Affiliations:** 1 Department of Endocrinology and Diabetology, Children’s Memorial Health Institute, Al. Dzieci Polskich 20, 04-730 Warsaw, Poland; 2 Department of Pathology, Children’s Memorial Health Institute, Al. Dzieci Polskich 20, 04-730 Warsaw, Poland; 3 Collegium Medicum, Jan Kochanowski University, Al. IX Wieków Kielc 19A, Kielce, Poland

**Keywords:** Hypopituitarism, Sellar, Children, Relapse, Progression

## Abstract

**Purpose:**

Craniopharyngioma is one of the most frequent benign tumours of the central nervous system in the paediatric population. Although it is a benign tumour according to the WHO classification, it significantly deteriorates the patient’s quality of life. The aim of this study is to assess if proliferation index Ki67 can be a useful marker of the risk of craniopharyngioma’s recurrence.

**Methods:**

Expression of Ki67 was examined in 85 specimens of primary craniopharyngioma and in 11 specimens of the recurring tumour. In all the cases, adamantinomatous type of craniopharyngioma was diagnosed. Values of Ki67 expression were compared between patients with and without recurrence, between patients with progression and relapse and between primary and recurrent tumours.

**Results:**

No statistically significant differences were found between proliferation index Ki67 values in tumours with recurrence and without (median values 2.5% and 3%, respectively, *p* = 0.69). The median value of proliferation index Ki67 in progression group was 1% and in the relapse group 4%; no statistical significance between those groups was found (*p* = 0.067). The median value of proliferation index Ki67 in primary tumours was 3% (0–20%) and in recurrent tumours it was 5% (0–14%). Despite the lack of statistical significance (*p* = 0.61), a tendency towards higher values of Ki67 in recurring tumours in comparison with primary tumours was shown.

**Conclusions:**

Proliferation index Ki67 is not a reliable prognostic factor of craniopharyngioma’s recurrence.

## Introduction

Craniopharyngioma is a benign, partially cystic epithelial tumour of the sellar region with two clinical and histopathological variants (adamantinomatous and papillary), which is considered to be of grade I malignancy according to WHO criteria [1]⁠.

Craniopharyngioma contributes to 2–5% of all intracranial tumours in the whole population [2]⁠ and 5.6–14.1% among children [3–6]⁠. Incidence rate is assessed in the whole population as 0.5–2/mln/year, on the average 1.3/mln/year [7–10]⁠. In children, craniopharyngioma is one of the most frequent benign tumours of the central nervous system (CNS) and contributes to approx. 56% of all cases of tumours of the sellar and suprasellar regions [9, 10]⁠.

Although craniopharyngioma is a histopathologically benign tumour according to the WHO classification [1], clinically, it is considered ‘partially malignant’. Aggressive behaviour of the tumour is reflected by its tendency towards damaging of adherent structures and an inclination for recurrence, invasive growth and higher mortality of patients suffering from it in comparison with the rest of the population [11]⁠.

Craniopharyngioma’s recurrence is one of the most serious problems for neurosurgeons, due to difficulty in predicting it and the high morbidity and mortality rates in removing recurrent tumours. Usually, recurrence rate is higher after partial resection (on average 71%) than after total resection (on average 21%), but it can be as low as 20% following partial resection and as high as 50% in case of total resection, as reported in some studies [12, 13].

This is the reason why the authors decided to make the subject of this paper an analysis of prognostic factors of craniopharyngioma’s recurrence.

Numerous studies are aimed at discriminating clinical, morphological, ultrastructural and immunohistochemical prognostic factors of recurrence of craniopharyngioma. Among immunohistochemical factors, use of proliferation index Ki67 was analysed [14–21]⁠, expression of proteins from p53 group (p63, p73) [13, 22–25] and the presence of growth hormone receptors was analysed [26–28]⁠, as well as IGF-1 receptors [27, 28]⁠, somatoliberin receptors, oestrogen receptors [14, 29]⁠, progesterone receptors [14, 29]⁠ and leptin receptors [26, 27]⁠, and furthermore the presence of Rosenthal fibres [30]⁠, microvascular density [31, 32]⁠, vascular endothelial growth factor (VEGF) [31–33], retinoic acid receptor (RAR) [34], cathepsins [34], matrix metalloproteinase 9 (MMP-9) [33], collagen IV [33], CXCL12/CXCR4 [35], Sonic Hedgehog signalling (SHH) pathway [36], SOX9 [37], CD166 [38], oncogenes BRAF/CTNNB1 [39]⁠, and β-catenin [38, 40, 41].

The authors of this study decided to investigate Ki67 proliferative index in craniopharyngioma. Ki67 is a nuclear antigen that shows expression in all phases of the cycle besides G0. All types of dividing cells show expression of Ki67 [42]⁠. There is no definite opinion on the usefulness of assessment of immunoexpression of proliferation index Ki67 in order to predict relapse or progression of craniopharyngioma. Contradictory data has been published. According to several authors, proliferation index Ki67 is not a prognostic factor of tumour’s relapse [15–19]⁠. Other researchers had contradictory findings, i.e. Nishi, Izumoto, Haba and Guadagno [14, 20, 38]⁠. However, the outcomes of one study demonstrated a statistically significant higher value of proliferation index Ki67 in relapsing tumours in comparison with primary tumours [17]⁠.

**Fig. 1:**
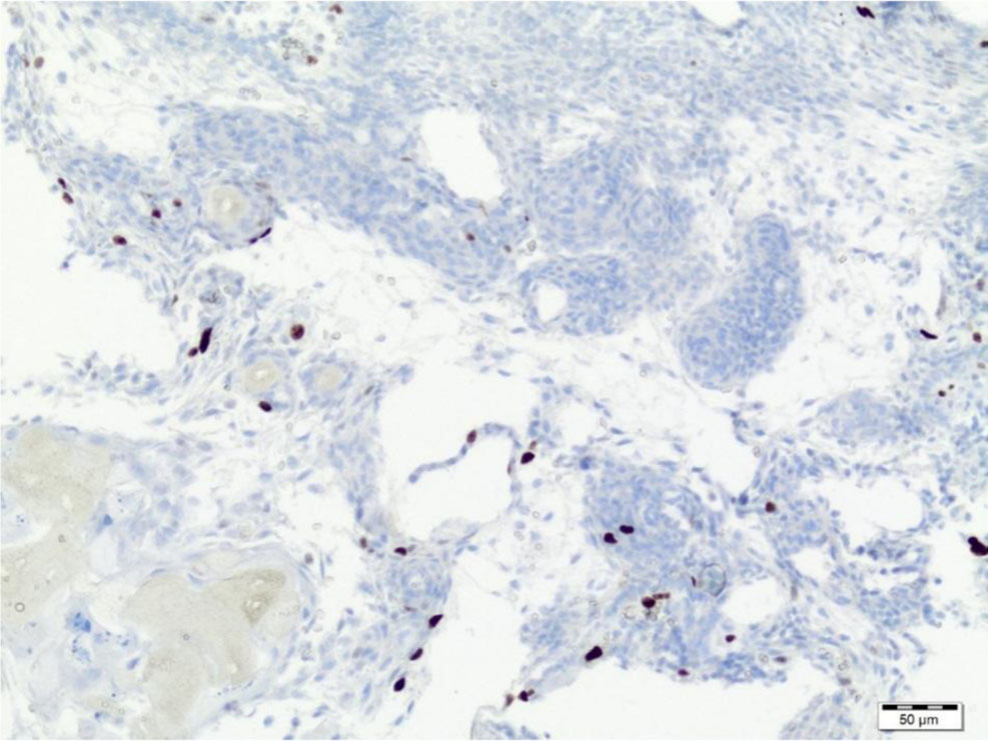
Low expression of Ki67 by immunohistochemistry in tumour cells (low magnification)

**Fig. 2:**
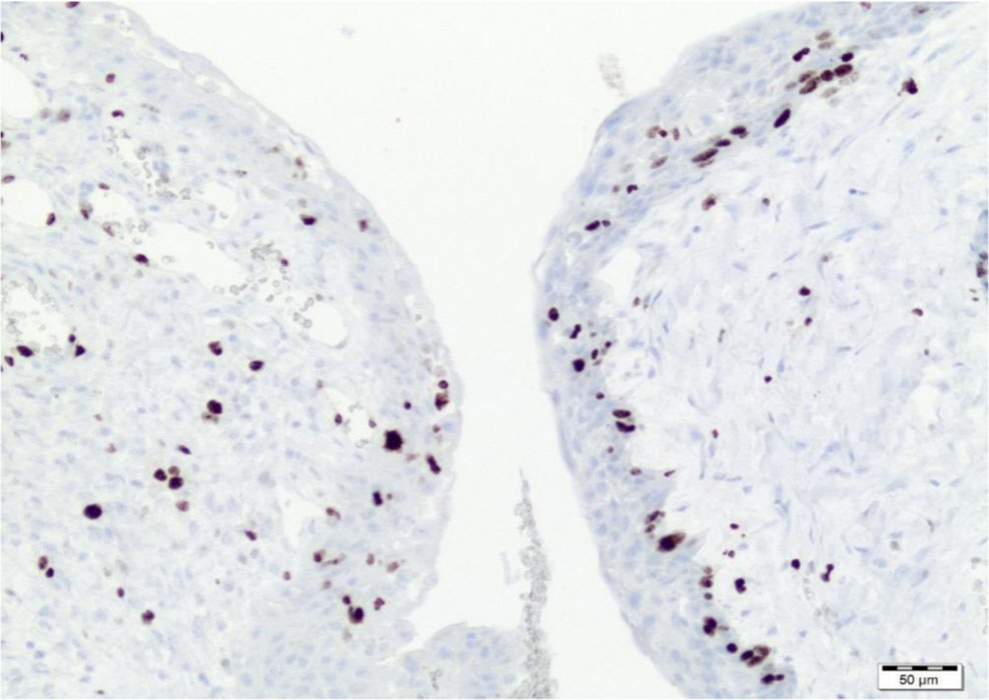
High expression of Ki67 by immunohistochemistry in tumour cells (high magnification)

Both attached photos present expression of marker Ki67 in histological slides counted in hot spots (Figs. 1 and 2). Specimens come from two different cases.

The added value of this research to previously published studies is that it took into consideration the value of proliferation index Ki67 in the most numerous and most homogeneous group of patients diagnosed with adamantinomatous type of craniopharyngioma analysed until now. What is more, the recurrence of the tumour was confirmed in imaging studies in all the cases. In the authors’ opinion, it is very valuable to validate the results of previous studies in this field in such a homogeneous group of patients.

## Methods

A retrospective analysis of histopathological material from tumours excised from 100 patients diagnosed with craniopharyngioma in Children’s Memorial Health Institute in Warsaw, Poland, between 1998 and 2012 was performed. Baseline characteristics of this group of patients are presented in Table 1.

**Table 1:** Baseline characteristics of the patients—84 cases

Characteristic	Value
Age at diagnosis (median/range) (years)	10.625 (0.08–21.8)
Sex (female/male)	42/42
Hypopituitarism* before operation	28 (33.3%)
Time from first symptoms to 1st operation (median/range) (years)	1.75 (0–10.58)
Tumour’s diameter (median/range) (mm)	35 (10–100)
Tumour’s localisation (suprasellar/intrasellar/both)	33/4/47 (39/5/56%)
Puget scale (0/1/2)	4/29/51 (5/35/60%)
Solid/cystic/mixed tumour	1/4/79 (1/5/94%)
Calcifications	69 (82%)
Craniotomy/transsphenoidal approach	79/5 (94/6%)
Partial/total resection	12/72 (14/86%)
Patients with progression/relapse	8/13 (9.5/15.5%)
Time from 1st operation to recurrence median (range) (years)	2.4 (0.25–8.34)
Reoperation due to recurrence	18 (21%)
Adjuvant radiotherapy after first operation	17 (20%)
Radiation dose (median/range) (cGy)	5325 (5000–5400)
Adjuvant chemotherapy	1 (1.2%)

Age of 83 patients <18, age of one patient 21.8 years

*Deficiency of 2 or more axes of pituitary hormones

Material from the tumours was fixed in 10% neutral buffered formalin and submitted to the Pathology Department of the Institute mentioned above. In 85 patients, histopathological examination was extended by the immunohistochemical marking. In 11 patients, Ki67 was assessed also in the secondary tumours (relapse/progression of the tumour).

The immunohistochemical reaction was performed using monoclonal antibodies against Ki67 (MIB-1 clone, company Dako). Proliferation index was measured by Ki67 marker in 3 areas of tumour tissue, which showed the highest proliferative activity. Nuclear staining in epithelial elements of tumour tissue was considered a positive reaction. The outcome was calculated as a percentage of positive reactions in 100 examined tumour cells. The highest outcome was taken into consideration for further analysis.

Recurrence was defined as either relapse or progression of the tumour. Relapse of the tumour was defined as the reappearance of the tumour after its total resection, which had been confirmed in MR/CT imaging performed after the operation. Progression was defined as an enlargement of the rest of the tumour left after a partial excision, with or without any clinical symptoms, but which required further treatment.

In statistical analysis, Student’s *t* test was used, and for characteristics of different than normal distribution non-parametric tests. Correlations between characteristics were analysed using test of independence *Χ*^2^ calculating correlation coefficient. The following values were calculated: arithmetic average, standard deviation, minimum and maximum values, median value and quartiles in graphs. *p* value accepted as a threshold for rejection of initial hypothesis was < 0.05. On box plots, the line inside the box visualises median value, top and bottom lines represent 1st and 3rd quartiles and ends of the whiskers represent minimum and maximum values observed. Outliers have been represented as individual dots.

The project was approved by the Institutional Review Board and Bioethics Committee.

## Results

In all the patients, adamantinomatous type of craniopharyngioma was diagnosed on the basis of histopathological examination.

**Fig. 3:**
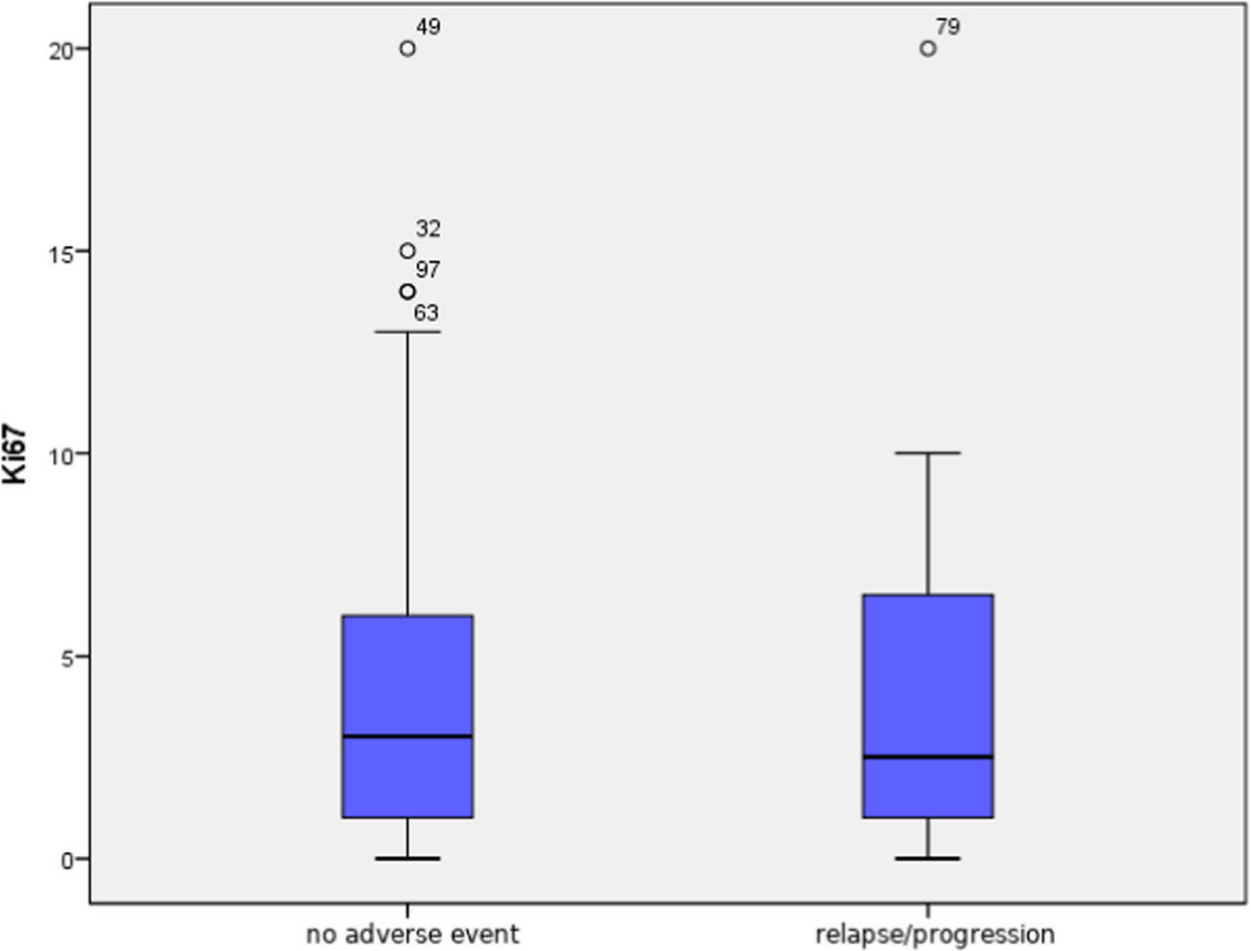
Comparison of proliferation index Ki67 value in patients without any adverse event (relapse/progression) and with relapse or progression

No statistically significant differences were found between value distribution of proliferation index Ki67 (*p* = 0.69) in specimens coming from patients who suffered from tumour relapse or progression in comparison with those in which such an event did not take place (median values 2.5% and 3%) (Fig. 3).

**Fig. 4:**
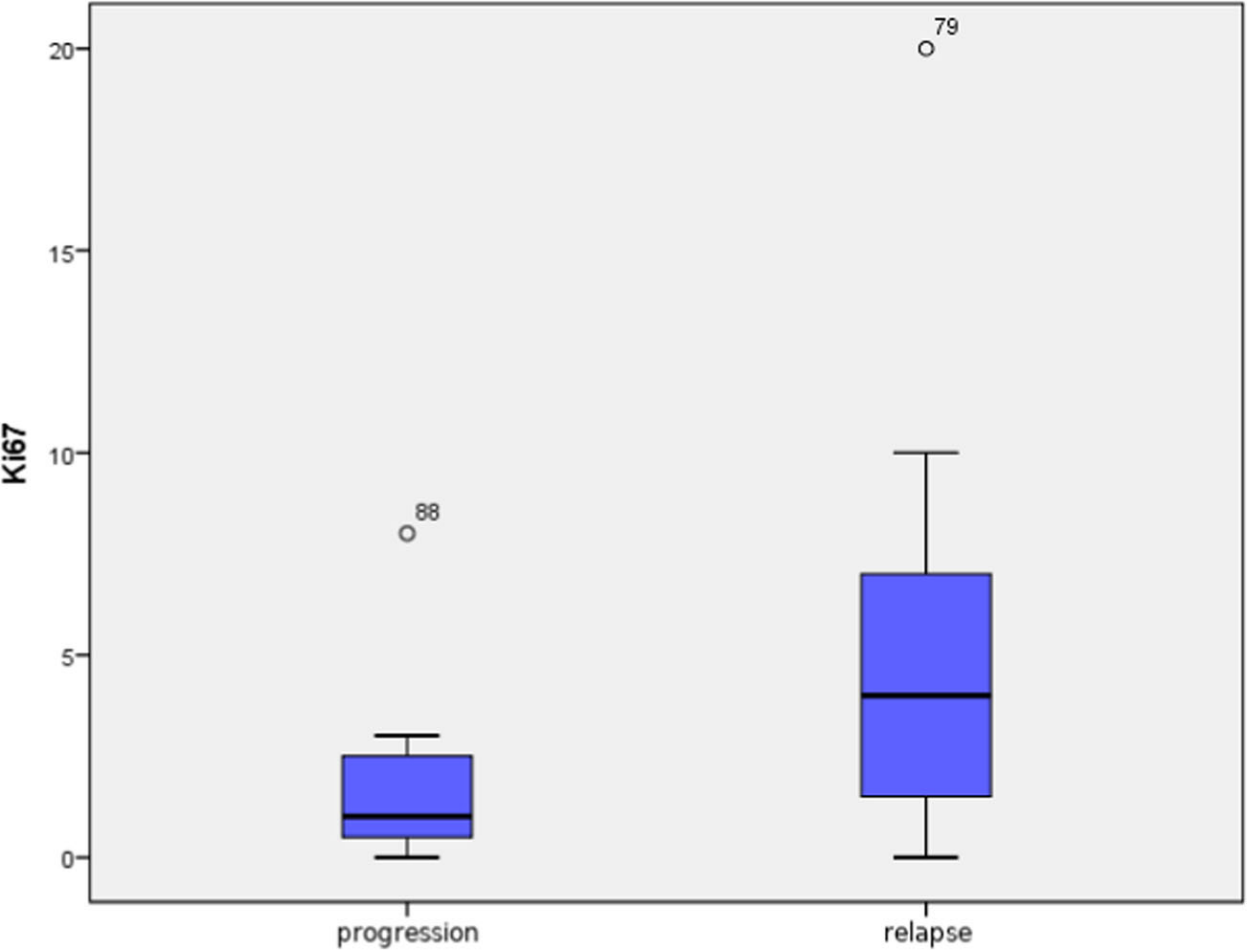
Comparison of proliferation index Ki67 value in patients with progression and with relapse

Although median value of proliferation index Ki67 differed significantly between the groups with progression and relapse (median value in progression group was 1% and in relapse group 4%), due to a large spread of outcomes, these differences were not statistically significant (*p* = 0.067). However, an evident tendency towards higher values of Ki67 was noticed in patients with relapse (Fig. 4).

**Fig. 5:**
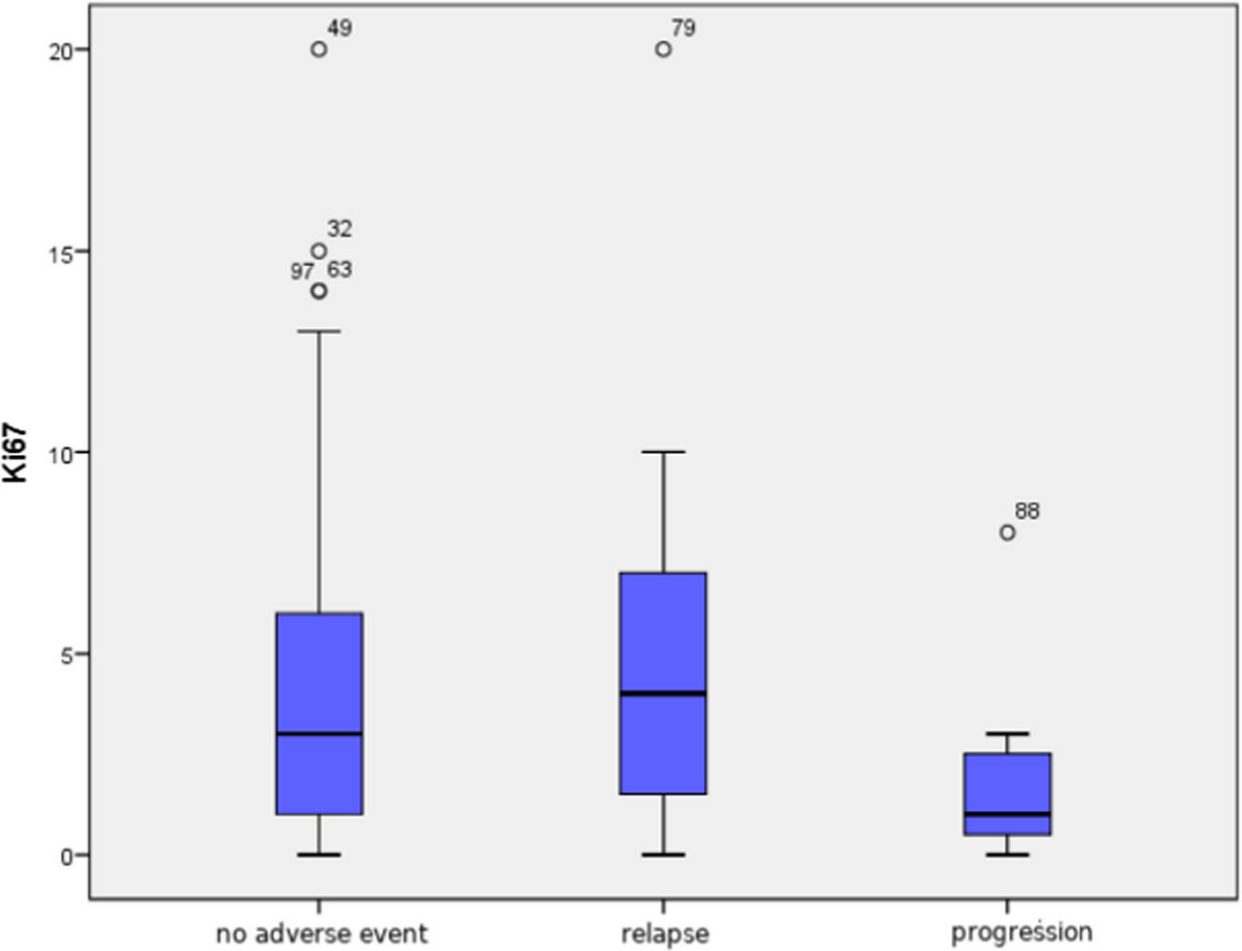
Comparison of proliferation index Ki67 value in tumours of patients with no adverse event, with relapse and with progression

No statistically significant difference in proliferation index Ki67 values was found between 3 analysed groups of patients: with no adverse event, with relapse and with progression (median values 2.5%, 4% and 1%, respectively, *p* = 0.127) (Fig. 5).

**Fig. 6:**
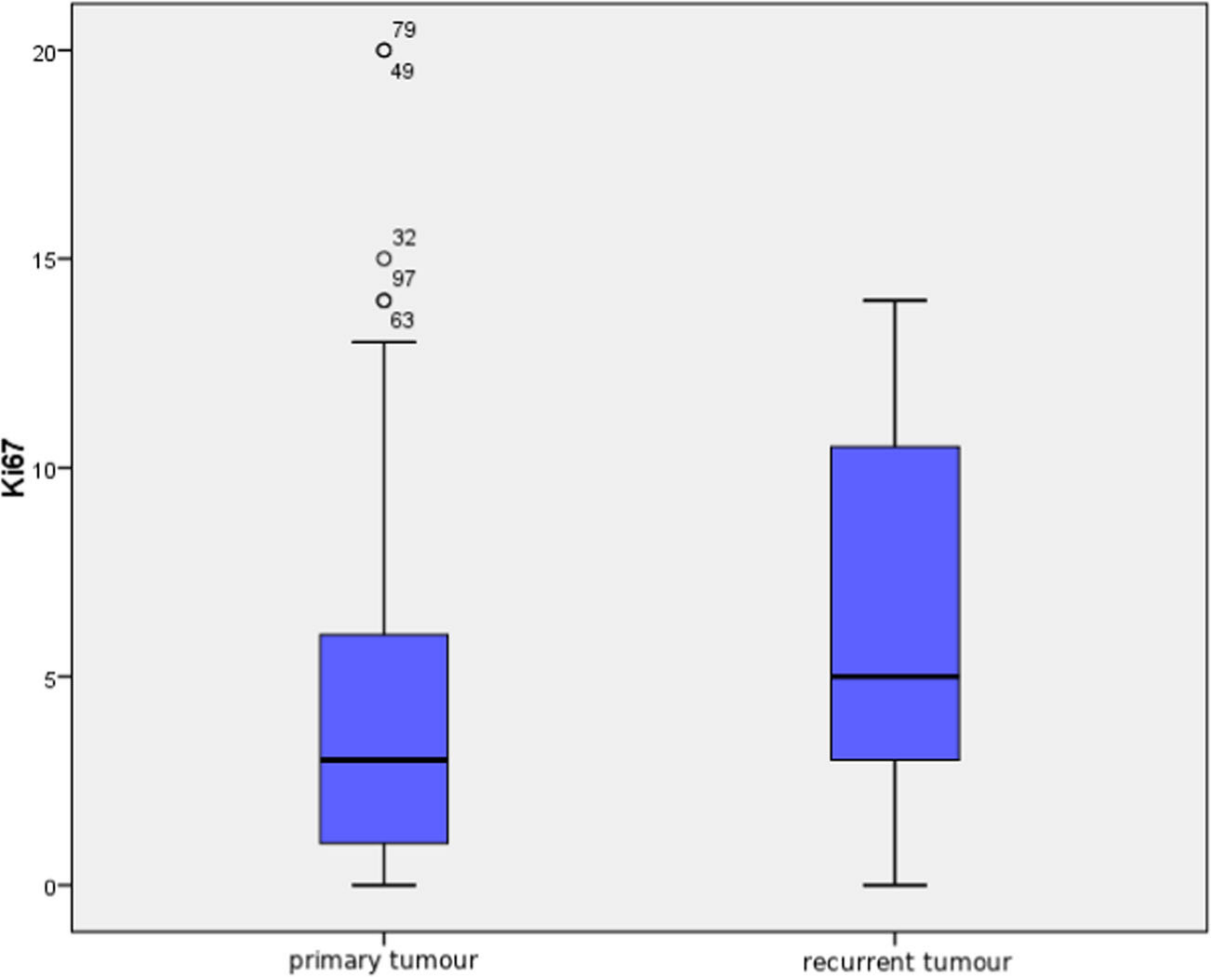
Comparison of proliferation index Ki67 in primary and recurrent tumours

As shown in Fig. 6, in spite of the lack of statistical significance (*p* = 0.61), there is a tendency towards higher values of Ki67 in recurring tumours. Median value in primary tumour was 3.0 (range 0–20%), and in recurring tumour 5.0 (0–14%). A statistically significant correlation was shown between Ki67 values in primary and recurrent tumours (*r* = 0.68, *p* = 0.044), meaning that if proliferation index in a primary tumour was high, higher values could be also expected in a recurring tumour.

## Discussion

Ki67 is an antigen, which is correlated in different types of neoplasms with proliferative activity of the tumour, its invasiveness and in some types of tumours also with prognosis [43]⁠.

Available literature provides researchers with ambiguous results concerning prognostic value of proliferation index Ki67 [Table 2.].

**Table 2:** Available literature on the subject of the study

Author	Number of all the patients/children	Age of patients (range, years)	Histopathological type of craniopharyngioma (aCP/pCP)	Median (or mean) Ki67 value (range) in primary tumours (%)	Was Ki67 considered a prognostic factor of CP recurrence?
Kim [11]	36/36	1–15	aCP	4.6 (1.1–13.5)	No
Izumoto [14]	43/NI	3–78	aCP/pCP	*	Yes
Losa [15]	47/16	6–78	aCP/pCP	8.6 (4.4–14)	No
Agozzino [16]	37/7	4 to > 40	aCP/pCP	22.12 (9–38.6)	No
Dickey [17]	40/NI	NI	aCP/pCP	NI (1.9–37)	No
Yalcin [18]	47/15	4–74	aCP/pCP	1.68 (0–12)	No
Raghavan [19]	37/15	3.7–59	aCP/pCP	7.8 (0.1–34.6)	No
Nishi [20]	17/13	5–46	aCP	10.84 (0.4–32.5)	Yes
Duo [21]	37/17	4–72	aCP/pCP	18 (1–49)	No
Guadagno [38]	41/7	2–77	aCP/pCP	6 (1–25)	Yes
Authors	84/84**	0.08–18	aCP	3 (0–20)	No

*NI* no information

*8% in patients with regrowth and 3.9% in patients without regrowth (no data about range of values)

**Age < 21.8 years

Outcomes of our own research, likewise reports of Losa, Dickey, Yalcin, Raghavan, Kim and Duo, did not demonstrate a statistically significant correlation between recurrence of craniopharyngioma and value of proliferation index Ki67 [11, 15, 17–19, 21]⁠. Different outcomes in this area were obtained only by Nishi, Izumoto and Guadano [14, 20, 38]⁠. Our results, as well as studies of Dickey, Raghavan and Kim, demonstrated higher values of proliferation index Ki67 in recurring tumours in comparison with primary ones [11, 17, 19]⁠. However, in only a study performed by Dickey the difference between Ki67 values was statistically significant [17]⁠. No difference between proliferation index Ki67 values in recurring and primary tumours was found by Agozzino and Yalcin [16, 18]⁠.

In analysed literature, which includes different groups of patients with respect to the number of patients (1–46), their age (children and adults) and histopathological type of craniopharyngioma, the range of values of proliferation index Ki67 in primary tumours was 1.68–22.12% (minimum value 0%, maximum value 49%), as presented in Table 2 [11, 14–21, 38].

Besides the authors’ study, there was only one study, performed by Kim et al., which concerned only the paediatric population with only an adamantinomatous type of craniopharyngioma [11]⁠. In the study by Nishi et al., only patients diagnosed with adamantinomatous craniopharyngioma were taken into consideration, but they were both children and adults. [20]. In the rest of the studies, both children and adults and different histopathological types of the tumour were taken into consideration.

The outcomes of studies of Kim et al. were consistent with those of our own research. Authors demonstrated similar mean values of Ki67 in primary tumours in groups with and without recurrence, 4.4% and 4.5% respectively, which confirmed that proliferation index Ki67 is not a prognostic factor of craniopharyngioma’s recurrence [11]. What’s more, a tendency towards higher values of proliferation index Ki67 in recurrent tumours in comparison with primary tumours was demonstrated, which is also coincidental with outcomes of our study [11]⁠.

Also, Yalcin et al. did not demonstrate that proliferation index Ki67 is a prognostic factor of recurrence of craniopharyngioma by analysing data of 47 patients diagnosed with both types of craniopharyngioma. Mean value of Ki67 in all the patients was 1.68%. Contradictory to our own research, no difference was demonstrated between values of Ki67 in primary and recurrent tumours. Furthermore, no statistically significant correlation was found between the value of proliferation index Ki67 and age, nor gender nor the histological type of the tumour [18]⁠.

Similar outcomes were also presented by Losa et al. who analysed data of 46 patients with adamantinomatous and papillary types of the tumour. The value of proliferation index Ki67 in patients with primary tumours without recurrence was 7.9%, and in patients with recurrence 9%, values did not differ significantly from the statistical point of view. The authors of this research did not state any correlation between Ki67 value and recurrence of the tumour, but a correlation between the high value of proliferation index Ki67 and presence of severe inflammation and diabetes insipidus was observed, which was not a subject of analysis in our own study [15].

Analogical were the outcomes of our study to those of Raghavan et al., which took into consideration 37 patients with both histopathological types of craniopharyngioma. The mean value of proliferation index Ki67 in primary tumours in children was 6.3%, in adults 8.9% and in children with a recurrence of the tumour 9.9%. It was stated that the value of proliferation index Ki67 was not a prognostic factor of recurrence of craniopharyngioma, but a tendency towards higher values of Ki67 was demonstrated in recurring tumours in comparison with primary tumours; however, the difference was not statistically significant, likewise in our study [19]⁠.

In a research performed by Dickey et al., which included 40 patients, no correlation was demonstrated between the recurrence of craniopharyngioma and the value of proliferation index Ki67. However, a statistically significant difference was shown between the values of Ki67 in primary and recurrent tumours [17].

Duo et al., in an analysis of 37 patients, demonstrated a lack of correlation between the recurrence of craniopharyngioma and the value of proliferation index Ki67. Furthermore, they demonstrated statistically significant higher mean values of proliferation index Ki67 in adults in comparison with children, respectively, 28.8% and 8.1%, with no correlation with the histopathological type of the tumour [21]⁠.

The mean value of proliferation index Ki67 in primary tumours in 37 patients in a study of Agozzino et al. was assessed to be 22.12%. In primary tumours with observed recurrence, the mean value of Ki67 was 27.5% and in tumours without recurrence 31.3%; no statistically significant difference was found between these outcomes [16].

Contradictory outcomes to ours were presented by Nishi, Izumoto and Guadagno [14, 20, 38]⁠.

Nishi et al. analysed values of proliferation index Ki67 in 17 patients with an adamantinomatous type of craniopharyngioma, and demonstrated that in patients without recurrence it was on average 3.4% and in patients with recurrent tumours it was significantly higher—13.2% on average. Moreover, it was demonstrated that in the majority (> 90%) of patients with recurring tumours, the value of proliferation index Ki67 was higher than 7% [20].

Outcomes similar to those presented above were obtained by Izumoto et al. who examined 43 patients, with both histopathological types of craniopharyngioma [14]. The value of the proliferation index Ki67 was statistically significantly higher in 15 patients with recurrence of the tumour in comparison with those without recurrence (7.8% towards 3.9%) [14].

In the study performed by Guadagno et al., Ki67 levels were measured in 41 craniopharyngiomas and a statistically significant association between high Ki67 (> 5%) and recurrences was found [38].

On the basis of outcomes of our study, accordingly to outcomes of the majority of cited authors, it has been stated that proliferation index Ki67 is not a prognostic factor of recurrence of craniopharyngioma.

Only 3 of the authors cited above, i.e. Nishi, Izumoto and Guadagno, stated that the value of proliferation index Ki67 is a prognostic factor of relapse and progression of craniopharyngioma [14, 20, 38]⁠.

Most of the authors agree on the fact that the value of proliferation index Ki67 in recurrent tumours is higher than in primary tumours, which was also confirmed by our study.

It needs underlining that hitherto published studies concerned small and heterogeneous groups of patients which included both children and adults, various histopathological variants of craniopharyngioma and various follow-up durations. What is more, various criteria of tumour’s recurrence were used (clinical and/or imaging). What is more, in the case of Ki67 assessment, high interobserver and interlaboratory variabilities have been reported, in part due to differences in staining methodologies, positivity thresholds and approaches to quantification. Against these reports, our own research that took into consideration 85 patients aged below 21.8 years diagnosed only with the adamantinomatous type of the tumour, the recurrence of the tumour confirmed in imaging studies and all the histopathological examinations were performed in one laboratory contributes to the most numerous and most homogeneous group of patients analysed until now.

Determination of proliferation index Ki67 may be legitimate in cases of suspicion of rare malignant transformation of craniopharyngioma or malignant de novo form. Especially vigilant oncological care and more radical treatment should be applied in the case of patients with high proliferation index Ki67 [44].

Although there is no clear evidence towards Ki67 prognostic value, a statement made by Chargari in 2007 seems to remain a reasonable approach, which is to take into consideration the value of proliferation index Ki67 as well as the presence of mass of the tumour in MR imaging, diameter of the primary tumour (higher progression risk in tumours > 4 cm) or cystic structure of the tumour while making the decision on the use of radiotherapy as adjuvant therapy [45]⁠.

Ambiguous outcomes of published studies concerning immunohistochemical prognostic factors including Ki67 indicate the need for further studies that would aim at identifying different prognostic factors of craniopharyngioma’s recurrence. Their selection would be exceptionally useful clinically during establishing of criteria of ordering adjuvant therapy after the partial operation and the rules of clinical follow-up of the patients.

Recent studies aimed at discriminating those factors indicate high expression in recurrent tumours of p53 tumour suppressor gene [13, 24, 25], the stem cell marker CD166 [38], chemokine receptor CXCR4 and its ligand CXCL12 [35] and also β-catenin accumulation [38, 40, 41].

Another of such factors can be the recently discovered immunomarker of mitotic activity of cancer cells—phospho-histone H3 (pHH3)—and the authors have also plans concerning its examination in our group of patients. The phosphorylation of histone H3 plays an important role in gene expression, chromatin remodelling, chromosome condensation and cell division. Prognostic significance of mitotic index using the mitosis marker anti-pHH3 antibody has been reported to be of great value in breast cancer, melanoma and meningioma. What is more, interpretation of pHH3 proliferative indices is more objective and significantly faster than that of Ki67 [46, 47].
